# ACU193: An Immunotherapeutic Poised to Test the Amyloid β Oligomer Hypothesis of Alzheimer’s Disease

**DOI:** 10.3389/fnins.2022.848215

**Published:** 2022-04-26

**Authors:** Grant A. Krafft, Jasna Jerecic, Eric Siemers, Erika N. Cline

**Affiliations:** Acumen Pharmaceuticals, Inc., Charlottesville, VA, United States

**Keywords:** Alzheimer’s disease, immunotherapy, oligomer-selective, therapeutic antibody, AβO = amyloid beta oligomer, synaptic plasticity

## Abstract

Alzheimer’s disease (AD) is an age-related neurodegenerative disease that affects 50 million people worldwide, with 10 million new cases occurring each year. The emotional and economic impacts of AD on patients and families are devastating. Approved treatments confer modest improvement in symptoms, and recently one treatment obtained accelerated approval from the United States Food and Drug Administration (FDA) and may have modest disease modifying benefit. Research over the past three decades has established a clear causal linkage between AD and elevated brain levels of amyloid β (Aβ) peptide, and substantial evidence now implicates soluble, non-fibrillar Aβ oligomers (AβOs) as the molecular assemblies directly responsible for AD-associated memory and cognitive failure and accompanying progressive neurodegeneration. The widely recognized linkage of elevated Aβ and AD spawned a comprehensive 20-year therapeutic campaign that focused primarily on two strategies – inhibition of the secretase enzymes responsible for Aβ production and clearance of Aβ peptide or amyloid plaques with Aβ-directed immunotherapeutics. Unfortunately, all clinical trials of secretase inhibitors were unsuccessful. Of the completed phase 3 immunotherapy programs, bapineuzumab (targeting amyloid plaque) and solanezumab (targeting Aβ monomers) were negative, and the crenezumab program (targeting Aβ monomers and to a small extent oligomers) was stopped for futility. Aducanumab (targeting amyloid plaques), which recently received FDA accelerated approval, had one positive and one negative phase 3 trial. More than 25 negative randomized clinical trials (RCTs) have evaluated Aβ-targeting therapeutics, yet none has directly evaluated whether selective blockage of disease-relevant AβOs can stop or reverse AD-associated cognitive decline. Here, we briefly summarize studies that establish the AD therapeutic rationale to target AβOs selectively, and we describe ACU193, the first AβO-selective immunotherapeutic to enter human clinical trials and the first positioned to test the AβO hypothesis of AD.

## Introduction

Alzheimer’s disease (AD) currently affects over 6 million people in the United States) and approximately 50 million people worldwide, and it is the sixth-leading cause of death in the United States (Alzheimer’s Association, 2021). Current demographic trends suggest that these numbers will triple by the year 2050, with associated healthcare costs expected to exceed $1.2 trillion in the United States alone, unless effective preventative measures or disease-modifying treatments emerge (Cahill, 2020; WHO, 2021). Four AD drugs (donepezil, rivastigmine, galantamine, and memantine) were approved by the FDA between 1996 and 2003 (New York Times, 1996; The Pharma Letter, 2000, 2001, 2003), with worldwide sales from 2015 to 2020 of $67B (BCC Research, 2020), yet these medications afforded only minimal palliative benefit to AD patients. In June 2021, following an 18-year gap in AD drug approvals, the FDA granted accelerated approval for Biogen’s therapeutic antibody aducanumab, which is currently marketed as Aduhelm at an annual wholesale cost of $28K per patient (Fleck, 2021; The Pharma Letter, 2021). In granting this approval, the FDA used the accelerated regulatory pathway rather than full approval. Full approval was nearly unanimously rejected by the Peripheral and Central Nervous System (PCNS) Drugs Advisory Committee, which concluded that the phase 3 clinical trial data did not provide sufficient evidence of efficacy (Alexander et al., 2021; Dunn, 2021; Restifo, 2021). Notwithstanding the approval of aducanumab, the circumstance remains that no medication with full approval addresses the underlying AD molecular pathology or prevents the inexorable progression of the disease. The field has now reached a general consensus that soluble, non-fibrillar amyloid beta (Aβ) oligomers (AβOs) are the primary neurotoxic molecular assemblies responsible for the cognitive dysfunction and progressive neurodegeneration that occur in AD (Lambert et al., 1998, 2001; Klein et al., 2001, 2004; Kirkitadze et al., 2002; Klein, 2002, 2006, 2013; Gong et al., 2003; Lacor et al., 2004, 2007; Walsh and Selkoe, 2004, 2007; De Felice et al., 2008; Viola et al., 2008; Krafft and Klein, 2010; Ondrejcak et al., 2010; Ferreira and Klein, 2011; Hayden and Teplow, 2013; Viola and Klein, 2015; Cline et al., 2018; Mroczko et al., 2018; Li and Selkoe, 2020; Tolar et al., 2021). In June 2021, ACU193 became the first AβO-selective therapeutic antibody to enter human clinical trials (Siemers, 2021), and the first candidate therapeutic poised to test the amyloid oligomer hypothesis (Lambert et al., 1998; Cline et al., 2018; Wang et al., 2018).

## The Current Status of Amyloid-Targeting Experimental Therapeutics

A comprehensive discussion of amyloid-directed AD therapeutics is beyond the scope of this paper; however, recent reviews have provided thorough coverage of this topic (Bullain and Doody, 2020; Avgerinos et al., 2021; Decourt et al., 2021; Jeremic et al., 2021; Lin et al., 2021; Restifo, 2021; Sun et al., 2021; Van Bokhoven et al., 2021; Xie et al., 2021; Yu et al., 2021). Jeremic et al. (2021) summarized the late-stage clinical failures of eight secretase inhibitors, and they report on the clinical status of eight amyloid-directed monoclonal antibodies. Decourt et al. (2021) provide concise summaries across a wider range of amyloid approaches, including secretase inhibitors and anti-amyloid antibodies. To varying degrees, the secretase inhibitors were effective in blocking conversion of amyloid precursor protein (APP) to Aβ peptide. Several studies in transgenic AD mice showed a treatment-related decrease in amyloid deposits (Deleye et al., 2017; Brendel et al., 2018), and one positron emission tomography (PET) study of AD patients treated with lanabecestat showed a significant treatment-related decrease in florbetapir-positive amyloid deposits (Wessels et al., 2020; Zimmer et al., 2021). Regardless of the outcome of PET imaging, clinical trial data did not reveal significant slowing of cognitive decline for any secretase inhibitor.

For more than a decade, clinical studies of amyloid-directed therapeutics have revealed varying pictures ranging from little or no reduction to substantial reduction of brain amyloid, depending on the target of the antibody. Avgerinos et al. (2021) has published a comprehensive review and meta-analysis of 17 amyloid-directed immunotherapeutic clinical trials. These authors concluded that the amyloid-directed therapeutics “induced clinical improvements of small effect sizes, biomarker improvements of large effect sizes, and increases in risk for the hallmark adverse event, amyloid-related imaging abnormalities (ARIA), by a large effect size, when all drugs were pooled together.” The first anti-amyloid immunotherapeutic tested in humans was bapineuzumab, which targeted amyloid plaque. A phase 2 trial showed comparable rates of cognitive decline for treated and placebo groups (Salloway et al., 2009), while an associated imaging study showed that bapineuzumab brought about only a very modest reduction of brain amyloid at the low antibody doses used in this early study (Rinne et al., 2010). Two large randomized phase 3 studies showed that bapineuzumab did not improve clinical outcomes in treated AD patients (Salloway et al., 2014). Solanezumab, which targets Aβ monomers, was the second anti-amyloid immunotherapeutic tested in humans. Two phase 3 studies (EXPEDITION and EXPEDITION2) did not achieve statistical significance on the primary outcome, but a small trend for slowing of progression was evident, with most secondary outcomes achieving nominal statistical significance (Doody et al., 2014). Florbetapir PET imaging of a subset of patients in the EXPEDITION3 study indicated that solanezumab did not reduce amyloid plaque (Honig et al., 2018).

Aducanumab, which targets primarily plaques and protofibrils (Sevigny et al., 2016), was evaluated in two phase 3 studies, ENGAGE and EMERGE. Both studies were terminated in 2019 after interim futility analysis of the ENGAGE study indicated that primary outcome goals would not be achieved (Arnold, 2020). Subsequent data analysis from EMERGE revealed slowing of cognitive decline by 18–27% over 18 months, leading Biogen to request regulatory approval from the FDA. Data from these studies have not yet been published in a peer-reviewed journal, however, the full data package is publicly available (FDA.gov, 2020). In November, 2020, the PCNS Drugs Advisory Committee to the FDA voted against aducanumab approval (Alexander et al., 2021; Dunn, 2021). Nevertheless, in June 2021, the FDA granted accelerated approval of aducanumab (Dunn et al., 2021). Writing on behalf of the FDA to explain this decision to PCNS committee members, Dr. Billy Dunn, Director, Office of Neuroscience at the FDA’s Center for Drug Evaluation offered the following: “*An effect on this surrogate endpoint must be shown to be reasonably likely to predict clinical benefit. We concluded that these requirements were met for aducanumab, with substantial evidence that the drug reduces amyloid beta plaque, and that this reduction is reasonably likely to predict clinical benefit*” (Dunn, 2021). In making its decision, the FDA considered recently published data from a phase 2 study of donanemab (Mintun et al., 2021), which showed statistically significant slowing of clinical progression based on the primary outcome measure, along with significant reduction in amyloid plaques. These data were not available to the PCNS Drugs Advisory Committee. A phase 3 study of donanemab is currently being conducted to demonstrate clinical benefit in a larger patient cohort (NCT05026866) ClinicalTrials.gov (2021). Donanemab binds to N-terminal pyroglutamate Aβ (AβpE3-42) which exists exclusively on amyloid plaques. A recent phase 1b florbetapir PET imaging study involving 61 AD patients demonstrated strong plaque-clearing ability (Lowe et al., 2021).

Lecanemab (BAN2401) is another anti-amyloid immunotherapeutic currently being evaluated in two phase 3 studies (Clarity AD, NCT03887455 and AHEAD 3-45, NCT04468659) (ClinicalTrials.gov, 2020). Lecanemab has been described as a protofibril-selective antibody, however, it exhibits appreciable binding to fibrillar Aβ (Gellerfors et al., 2009) and it also binds high molecular weight (80–2000 kDa) AβOs, but not lower molecular weight AβOs (dimer to 18-mer) (Sehlin et al., 2012; Yang et al., 2013). The lecanemab phase 2 study did not achieve statistical significance on its primary outcome measure at 12-months. However, 18-month Bayesian and frequentist analyses demonstrated that brain amyloid reduction was accompanied by modest slowing of cognitive decline across several clinical endpoints (Swanson et al., 2021).

Gantenerumab is another amyloid-directed immunotherapeutic that reduced brain amyloid substantially (Klein et al., 2019, 2020), however, interim futility analysis of the corresponding phase 3 cognitive data showed that the treated and placebo groups were not significantly different (Ostrowitzki et al., 2017). The phase 3 trial of gantenerumab at higher doses has resumed (NCT03444870).

Safety is a significant issue and potential liability for all current plaque-targeting immunotherapeutics because they all cause amyloid-related imaging artifacts (ARIA), which are indicative of cerebral edema (ARIA-E) or microhemorrhage (ARIA-H). ARIA-E in particular may be problematic; symptoms can include headache, confusion, dizziness, nausea, fatigue, visual impairment, blurred vision, and gait disturbance (Salloway et al., 2021). A significantly increased risk of ARIA has been observed for carriers the apolipoprotein E (ApoE) ε4 allele. In phase 3 aducanumab studies, for patients taking aducanumab, ARIA-E was seen in 43% of ApoE ε4 carriers and 20.3% of non-carriers (Salloway et al., 2021). Gantenerumab was associated with ARIA-E in 10.7% of ε4 homozygotes, 5.4% of ε4 heterozygotes, and 1.8% of ApoE ε4 non-carriers (Ostrowitzki et al., 2017) using a dose lower than that being studied currently. Lecanemab triggered ARIA-E in 14.3% of patients who were ApoE ε4 carriers and 8.0% in patients who were non-carriers for patients taking 10 mg/kg every 2 weeks (Swanson et al., 2021). In a phase 2 study of donanemab, ARIA-E occurred in 44% of patients taking donanemab who were ApoE ε4 homozygotes, 30% of patients who were heterozygotes, and 11% of patients who were non-carriers (Mintun et al., 2021).

Concern about treatment-emergent ARIA has led to the recommendation of surveillance magnetic resonance imaging (MRI) scans in labeling for aducanumab and based on recommendations from independent experts. The current aducanumab package insert in the United States recommends surveillance MRI scans prior to the 7th and 12th infusions. An independent panel of experts has developed “appropriate use criteria” which includes a recommendation for surveillance MRIs prior to the 5th, 7th, and 12th infusions (Cummings et al., 2021a). Additionally, an MRI should be obtained whenever symptoms suggestive of ARIA occur. Thus, treatments associated with ARIA require additional monitoring by MRI scans, resulting in greater complexity and costs associated with these treatments.

The expectation that reduction of brain amyloid should confer clinical benefit follows directly from the amyloid cascade hypothesis (ACH) and its core tenet that “deposition of amyloid β protein” is the cause of AD (Hardy and Higgins, 1992). Because much of the discovery and development effort leading to anti-amyloid therapeutics was based on the ACH, it was widely anticipated that reducing brain amyloid to undetectable levels would *stop* cognitive decline. Yet, over the dozen years between publication of the bapineuzumab phase 2 trial (Salloway et al., 2009) and aducanumab’s FDA accelerated approval (The Pharma Letter, 2021), the best outcome associated with substantial amyloid reduction was 18–27% slowing of cognitive decline by aducanumab in the EMERGE study (Cummings et al., 2021b), and approximately 30–35% slowing in the phase 2 donanemab study (Mintun et al., 2021). As noted elsewhere, therapies that target plaque and reduce it are associated with ARIA, which diminishes the clinical utility of these drugs.

### Implications of Treatments Targeting Oligomers for Other Amyloid-Related Therapies

Testing the oligomer hypothesis is certainly an important goal for the field, however, from a broader perspective, the ACH will continue to be debated. One might pose the question as to whether any future negative trial would nullify the ACH. As detailed elsewhere in this manuscript, the various therapeutic approaches generally considered relevant to the ACH operate by mechanisms that have clear differences. Small molecule inhibitors of gamma-secretase and BACE were unsuccessful, and in fact, caused slight cognitive worsening. Monoclonal antibodies have generated more promising results in certain cohorts, but even these therapeutics have important differences and should not be considered a monolithic class. For example, solanezumab targets monomers rather than plaque, does not reduce plaque and does not cause ARIA. Aducanumab and donanemab target and reduce amyloid plaques based on PET imaging, however, they cause ARIA as a result. Lecanemab may target protofibrils preferentially compared with monomers, however, it also exhibits appreciable binding to amyloid fibrils (Englund et al., 2007; Lord et al., 2009; Magnusson et al., 2013). As expected, plaque reduction and ARIA are observed, though existing data indicate that the ARIA incidence may be less than that observed for aducanumab and donanemab treatments (Logovinsky et al., 2016). As discussed in the section “ACU193: AD Immunotherapy that Selectively Targets Amyloid β Oligomers,” plaque binding and ARIA are not expected for ACU193, based on its binding selectivity for AβOs; however, clinical data confirming this expectation are not yet available. Thus, it is important to recognize important differences in mechanism of action of the varied therapeutics presumed to test ACH. Over time, failed clinical trials may result in formulation of new or modified hypotheses, which eventually become accepted when supporting clinical data emerge.

### Recent Advances in Trial Design in Alzheimer’s Disease Studies

An important advance in a number of therapeutic antibody trials has been the inclusion of individuals assessed to have early AD i.e. mild cognitive impairment (MCI) or mild dementia due to AD. In the past, some have speculated that any amyloid-related treatment would need to be given as secondary prevention to individuals who are cognitively normal but harbor amyloid plaques. Such studies include public–private partnerships such as the Anti-Amyloid Treatment in Asymptomatic Alzheimer’s (A4) study and the AHEAD 3-45 study. By definition, these studies are conducted with cognitively normal individuals and require very sensitive cognitive measure. Trial design parameters for these secondary prevention studies are not particularly well understood, so while prevention may be a worthy goal, negative trial results may emerge for a therapeutic that could be efficacious in a symptomatic population. The recent emergence of promising efficacy signals in certain early AD clinical cohorts receiving high doses of aducanumab, donanemab (Mintun et al., 2021), and lecanemab (Siemers et al., 2022) is an encouraging development.

## The Rationale for Clinical Development of an Amyloid β Oligomer-Selective Therapeutic

While extensive plaque reduction may confer some clinical benefit, these treatments will be associated with ARIA, such that efficacy might be achieved at the cost of significant safety concerns. Ideally, an AD therapeutic must intercept the most relevant mechanistic target, and it must possess properties that enable efficient and selective interaction with that target. An AβO-selective therapeutic may represent such an approach to the treatment of AD.

### The Role of Deposited Amyloid Plaques in the Pathogenesis of Alzheimer’s Disease

The near-complete failure of anti-plaque therapeutics suggests that amyloid plaques and deposits are not the ideal target for AD therapeutics. Critics of the ACH have argued that deposition of Aβ protein is not the cause of AD (Lee et al., 2004; Teich and Arancio, 2012; Mullane and Williams, 2013, 2018; Morris et al., 2014, 2018; Behl, 2017; Makin, 2018; Panza et al., 2019; Høilund-Carlsen et al., 2020; Tolar et al., 2020). To be fair, however, the ACH relied heavily on compelling and irrefutable evidence that early-onset AD-causative mutations increased production of Aβ42, the most abundant plaque protein (Hardy and Allsop, 1991; Hardy and Higgins, 1992). The obvious inference made by Hardy was that elevated Aβ42 monomer deposited into plaques, which initiated the damage that occurs in AD. What Hardy and colleagues could not have known or anticipated in the early 1990s, was that Aβ42 monomer was capable of self-assembly into soluble, non-fibrillar neurotoxic oligomers possessing the ability to interfere directly with synaptic function and learning and memory.

That scenario came to light only a few years after the ACH was published, when Oda et al. (1995) observed soluble, neurotoxic Aβ42 structures in incubations of Aβ42 with small amounts of apoJ. Aβ42 fibril formation was unexpectedly inhibited, and instead, highly neurotoxic supernatant solutions were formed. Lambert et al. (1998) carried out extensive follow-up studies involving atomic force microscopy (AFM) and gel analysis to characterize soluble non-fibrillar oligomeric structures, ranging in size from trimer to dodecamer (Krafft et al., 1998). The soluble, globular oligomers exhibited potent neurotoxicity in hippocampal brain slice cultures, and at lower concentrations, the ability to block long-term potentiation (LTP) when added to organotypic brain slice cultures or injected into anesthetized rats. These neurotoxic oligomers were referred to as amyloid β-derived diffusible ligands (ADDLs). Lambert et al. (1998). wrote: “*We hypothesize that impaired synaptic plasticity and associated memory dysfunction during early stage Alzheimer’s disease and severe cellular degeneration and dementia during end stage could be caused by the biphasic impact of Aβ-derived diffusible ligands acting upon particular neural signal transduction pathways*.”

Further studies demonstrated the ability to generate rabbit polyclonal antibodies exhibiting > 80:1 AβO:monomer selectivity, and the polyclonal serum was used to detect AβOs in homogenates from AD frontal cortex and temporal cortex, but not in cerebellar homogenates (Lambert et al., 2001). The polyclonal serum also exhibited dose-dependent blockage of AβO toxicity in a PC12/MTT assay, a rudimentary demonstration of an AβO-selective immunotherapeutic approach.

### Two Decades of Profiling Bolsters the Amyloid β Oligomer Hypothesis

In 2001, Klein et al. reiterated the AβO hypothesis and argued the case for targeting Aβ oligomers. In 2002, Kirkitadze et al. recognized the emergence of AβOs as a clear paradigm shift, and Walsh et al. (2002a) emphasized the potential importance of AβOs as a therapeutic target. In the subsequent two decades, many studies across multiple disciplines have gathered extensive data that implicate AβOs as the causative AD structures, as discussed in a number of comprehensive reviews (Klein, 2002, 2006, 2013; Walsh and Selkoe, 2004, 2007; Viola et al., 2008; Krafft and Klein, 2010; Ondrejcak et al., 2010; Ferreira and Klein, 2011; Hayden and Teplow, 2013; Ferreira et al., 2015; Viola and Klein, 2015; Cline et al., 2018; Mroczko et al., 2018; Li and Selkoe, 2020; Tolar et al., 2021).

Several distinct AβO activities and characteristics are particularly noteworthy. AβOs bind with high affinity to a subset of hippocampal and cortical neurons (Lacor et al., 2004, 2007; Barghorn et al., 2005; Koffie et al., 2009; Shughrue et al., 2010; Pickett et al., 2016; Wang et al., 2017), indicative of specific binding to discrete cell surface receptors that are developmentally regulated. In rodent hippocampal slice preparations, AβOs rapidly inhibit LTP (Lambert et al., 1998; Walsh et al., 2002b; Wang et al., 2002; Rowan et al., 2004; Barghorn et al., 2005; Rammes et al., 2011). Direct injection of AβO solutions into the rodent brain leads to reversible impairment of cognitive function (Cleary et al., 2005; Townsend et al., 2006; Poling et al., 2008; Reed et al., 2011) and injection of AβO solutions into the lateral cerebral ventricle of non-human primates leads to formation of neurofibrillary tangles (Forny-Germano et al., 2014) and synapse loss (Batista et al., 2018). Recently, ultra-high resolution microscopy revealed that AβOs do not bind directly at the synaptic cleft, but instead, form distinct nanoscale clusters encircling the postsynaptic membrane (Actor-Engel et al., 2021). Significant binding was also observed at presynaptic axon terminals. This study further demonstrated that LTP was impaired only at AβO-targeted synapses and not at neighboring AβO-free synapses. Importantly, aberrant AβO signaling activates GSK3β-mediated hyperphosphorylation of tau (De Felice et al., 2008; Zempel et al., 2010; Ochalek et al., 2017), which eventually causes tau aggregation, paired helical filament formation, cytoskeletal collapse, and neurodegeneration. Because AβOs appear to act upstream of tau abnormalities and inhibit LTP rapidly after binding, therapies that target aggregated, hyperphosphorylated tau or GSK3β would not be expected to rescue neurons from early LTP compromise that directly and immediately compromises information storage, well before any tau dysfunction occurs.

Many studies have identified interactions between AβOs and a variety of cellular proteins, however, the identity of the receptor(s) that mediate peri-synaptic AβO binding, aberrant AβO signaling and disrupted synaptic plasticity remains to be established (Laurén et al., 2009; Benilova and De Strooper, 2013; Xia et al., 2016; Ding et al., 2019; Smith et al., 2019; Guo et al., 2020; Lao et al., 2021). The review by Mroczko et al. (2018) provides a succinct summary of many cell surface molecules for which AβOs have apparent affinity. The principal conundrum for many of the putative receptor candidates is a relatively high measured equilibrium dissociation constant (Kd) for the binding interaction between AβOs and the putative receptor.

Amyloid β oligomers are widely reported to be the most neurotoxic form of Aβ, capable of triggering aberrant neuronal signaling which leads to loss of synapses and progressive neurodegeneration (Gong et al., 2003; De Felice et al., 2008; Krafft and Klein, 2010; Ondrejcak et al., 2010; Zempel et al., 2010; Klyubin et al., 2012; Koffie et al., 2012; Hayden and Teplow, 2013; Hefti et al., 2013; Fowler et al., 2014; Goure et al., 2014; Batista et al., 2018; Hong et al., 2018). Wang et al. (2018) described the dysregulation of calcium homeostasis in cultured neurons by 150–180 pM AβOs, the most potent AβO signaling yet reported. Size exclusion chromatography (SEC) analysis of these AβO solutions revealed a predominant peak eluting shortly before the 43 kDa standard, likely corresponding to the 56 kDa dodecamer identified in human AD brain extracts by 2D gel analysis, and frequently observed in synthetic AβO preparations (Gong et al., 2003; Bernstein et al., 2009; Ahmed et al., 2010; Inayathullah and Teplow, 2011; Bisceglia et al., 2018). Lesné et al. (2006) also isolated stable dodecamer (referred to as Aβ*56) from transgenic AD mouse brain extracts, and showed that its appearance correlates closely with cognitive deficits in three different lines of transgenic AD mice (Billings et al., 2007; Cheng et al., 2007).

The compiled experimental evidence implicating AβOs as the cause of AD memory malfunction and neurodegeneration is strong and continues to gain support. Perhaps the strongest support comes from discovery of an inherited APP mutation (E693Δ), known as the Osaka mutation (Tomiyama et al., 2008; Inayathullah and Teplow, 2011), which results in production of an Aβ peptide variant (Aβ E22Δ) lacking glutamic acid residue 22. This Aβ variant possesses the surprising properties of enhanced oligomerization and no fibrillization. Even though the APP E693Δ mutation reduces Aβ secretion by 38%, the enhanced oligomerization of Aβ E22Δ brings about memory loss in affected family members at ages as young as 35 (Tomiyama and Shimada, 2020). PET imaging of Osaka carriers shows very little deposited amyloid, while post-mortem characterization shows extensive tau pathology and neurofibrillary tangles, with few or no amyloid plaques. In other words, AD without amyloid plaques, coupled with an oligomerization-prone Aβ variant, plainly implicates AβOs as the relevant AD neurotoxins.

## ACU193: Alzheimer’s Disease Immunotherapy That Selectively Targets Amyloid β Oligomers

**FIGURE 1:**
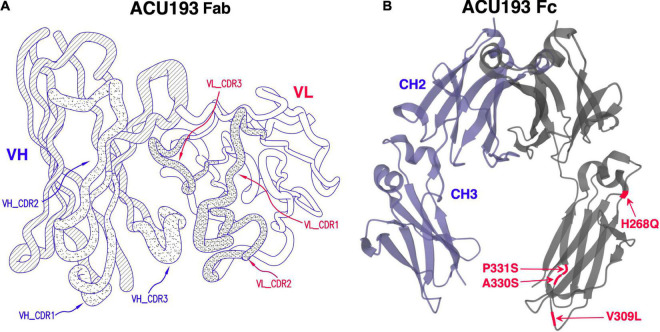
**(A)** A three dimensional model of the ACU193 Fab, depicting the heavy and light variable regions with locations of the CDRs, the sequences of which have been published (Goure et al., 2015, 2016; Gaspar et al., 2016). **(B)** A three dimensional model of the IgG2m4 Fc region. The four amino acid changes (highlighted in red) reduce complement activation and antibody-dependent cellular cytotoxicity (An et al., 2009; Strohl, 2010).

**FIGURE 2:**
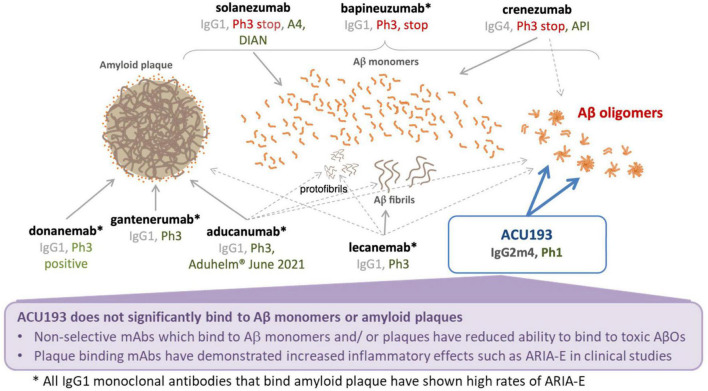
Summary comparison of ACU193 to amyloid-directed therapeutic antibodies in clinical development.

ACU193 is a humanized, affinity-matured, immunoglobulin G2m4 (IgG2m4) subclass monoclonal antibody, derived from the murine immunoglobulin G1 (IgG1) parent, ACU3B3 (Acton et al., 2014). The sequence of ACU193, which was first designated as clone 19.3, has been published (Goure et al., 2015, 2016). Figure 1A provides a 3D model of the ACU193 Fab region, highlighting the relative locations of the complementarity determining regions (CDR1, CDR2, and CDR3) in the variable light (VL) and variable heavy (VH) chains. The IgG2m4 isotype incorporates four amino acid changes (H268Q, V309L, A330S, and P331S) within the C1q binding region to reduce complement activation and antibody-dependent cellular cytotoxicity (An et al., 2009; Strohl, 2010). Figure 1B illustrates a 3D model of the IgG2m4 Fc region, and it highlights the location of the four engineered mutations. As discussed in more detail below, ACU193 binds with high selectivity to soluble AβOs versus Aβ monomer (section “Selectivity of ACU193 for Amyloid β Oligomers Versus Monomeric Amyloid β”), and it exhibits little or no binding to plaques or diffuse Aβ deposits (section “Selectivity of ACU193 for Amyloid β Oligomers Versus Amyloid Plaques”). This characteristic significantly distinguishes it from other therapeutic monoclonal antibodies that primarily bind Aβ monomers or fibrillar forms of Aβ. ACU193 binding to AβOs prevents synaptic attack, thereby allowing neurons to resume normal signaling. Because it selectively targets AβOs, ACU193 is expected to provide clinical benefit and safety superior to anti-Aβ antibodies currently in clinical development, as depicted in Figure 2.

### Selectivity of ACU193 for Amyloid β Oligomers Versus Monomeric Amyloid β

**FIGURE 3:**
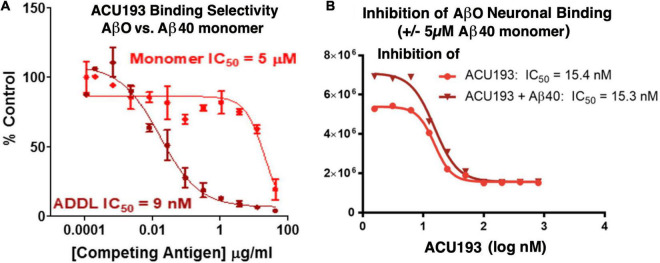
**(A)** Competitive ELISA for ACU193 binding to AβO or monomeric Aβ_40_ (Savage et al., 2014); **(B)** binding affinity of ACU193 to AβOs does not change significantly when 5 μM monomeric Aβ40 is added to the cell culture medium. These results support the conclusion that selectivity of ACU193 for AβOs is maintained in a biochemical environment simulating the brain (for methods, see Shughrue et al., 2010). Experiments in both figures use ADDLs (i.e., amyloid-derived diffusible ligands (Lambert et al., 1998; Chromy et al., 2003) as the synthetic AβO model system.

ACU193 exhibits significant preferential binding to AβOs over Aβ monomers. In a competition ELISA assay (Figure 3A), ACU193 binds to AβOs with 650-fold greater affinity than to Aβ monomers (Savage et al., 2014; Goure et al., 2016). Because this value does not account for the larger average size of synthetic AβOs used in these experiments, on a molar basis, the selectivity may be as high as 10,000-fold. Further evidence of ACU193 selectivity for AβOs was obtained in a cell-based AβO binding assay in which a very high concentration (5 μM) of monomeric Aβ did not decrease the ACU193 binding affinity for AβOs (Figure 3B). ACU193’s selectivity for AβOs in the presence of abundant Aβ monomers is expected to be representative of the *in vivo* levels of these Aβ species in AD patients. Thus, ACU193 does not experience “target distraction” from non-toxic Aβ monomers in an environment simulating brain interstitial fluid.

### Selectivity of ACU193 for Amyloid β Oligomers Versus Amyloid Plaques

**FIGURE 4:**
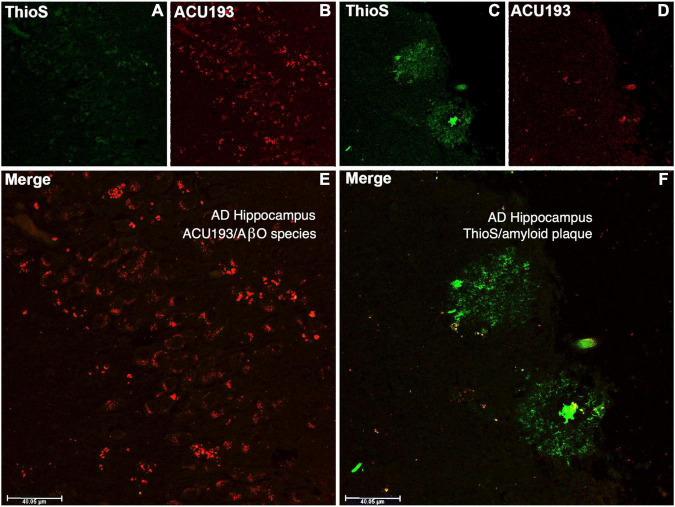
The upper left portion of the immunohistochemistry figure shows that in areas of advanced AD hippocampal tissue with no amyloid plaque binding (no green fluorescent ThioS staining, **(A)** there is substantial ACU193 binding (red fluorescent staining, **(B)** not related to amyloid plaque. The merge of these panel **(E)** shows ACU193 binding with no amyloid plaque present. On the upper right portion of the figure, the area that is positive for amyloid plaque (green fluorescent staining, **(C)** shows minimal ACU193 binding (red fluorescent staining, **(D)**. The merge of these panel **(F)** shows the minimal binding of ACU193 (red fluorescent staining) at the periphery of the amyloid plaque (green fluorescent staining), most likely related to AβOs binding at or associating with the periphery of the amyloid plaque.

ACU193 binds AβOs from CSF and brain tissue extracts of AD patients, and it exhibits minimal or no binding to amyloid plaques, as illustrated in Figure 4. Amyloid plaques stained with thioflavin S appear fluorescent green, while the binding of ACU193 appears as red fluorescence (Cline et al., 2019). Substantial ACU193 binding can be observed in regions that are without thioflavin S-stained amyloid plaques (Figures 4B,E), with minimal binding in regions exhibiting thioflavin-S-positive fibrillar Aβ structures (Figure 4D). Close examination does reveal occasional co-localization of ACU193 at the periphery of some amyloid plaques (Figure 4F). It is likely that this ACU193 localization reflects binding to AβOs associated with plaque surfaces (Gaspar et al., 2016). Taken together, these results are consistent with the concept that ACU193 binds endogenous AβOs, does not prevent thioflavin-S binding to plaques, and importantly, preferentially binds AβOs versus plaque-associated Aβ.

**FIGURE 5:**
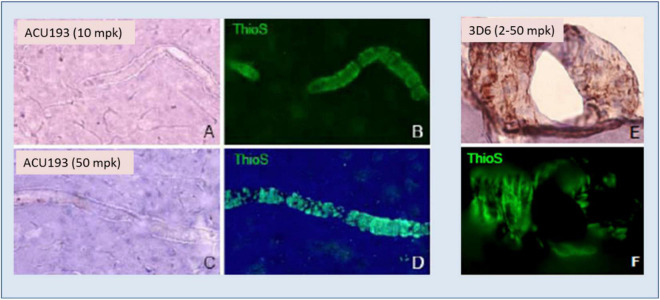
ACU193 **(A,C)** shows no binding to the vascular amyloid that is visible in the vessels stained by thioflavin-S (ThioS, green fluorescence, **B,D**) in the brain 24 h following IV dosing of 10 or 50 mg/kg (mpk) in 7- to 8-month-old Tg2576 mice. In contrast, hu3D6 (bapineuzumab) binds vascular amyloid **(E,F)** at all dose levels assessed (Krafft et al., 2012).

ACU193 exhibited no binding to amyloid deposits surrounding blood vessels (cerebral amyloid angiopathy) in AD brain slices, nor did it bind to vascular amyloid when injected into transgenic mice, as illustrated in Figure 5. On the other hand, injected bapineuzumab bound to vascular amyloid at all doses tested (Figures 5E,F).

In view of the linkage between the amyloid plaque-binding properties of multiple amyloid-directed antibodies and the frequent occurrence of ARIA (e.g., aducanumab, gantenerumab, lecanemab, and donanemab), the negligible binding of ACU193 to amyloid plaques, including amyloid plaques associated with cerebral amyloid angiopathy, suggests that ACU193 treatment will not elicit ARIA side effects.

### ACU193 Binds to a Broad Molecular Weight Range of Amyloid β Oligomers

**FIGURE 6:**
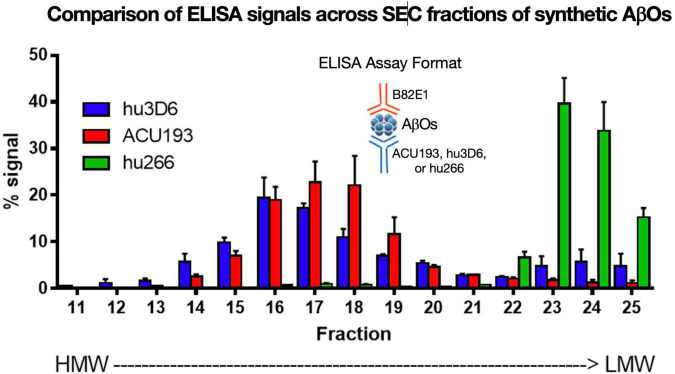
Binding of humanized antibodies to SEC fractions of synthetic AβOs (ADDLs – Lambert et al., 1998; Chromy et al., 2003; Hepler et al., 2006). SEC fractionation of AβOs with sandwich ELISA detection (Savage et al., 2014). Hu3D6 is also known as bapineuzumab; hu266 is also known as solanezumab. These data demonstrate the specificity of ACU193 for oligomers versus monomers, and also demonstrate the ACU193 is capable of binding AβOs ranging from dimers to higher order structures up to several hundred kDa.

ACU193 binds a broad spectrum of AβOs across various molecular weights. AβOs prepared from synthetic Aβ42 were fractionated by SEC (Figure 6) and characterized by ELISA using ACU193, hu3D6 (bapineuzumab) or hu266 (solanezumab) as the capture antibody and biotinylated anti-human Aβ antibody 82E1 for detection (Goure et al., 2015). These data show ACU193 binds mid- to higher molecular weight AβOs, with preferential binding to mid-molecular weight oligomers compared to hu266. This range of molecular weights includes dodecameric AβOs that are thought to be physiologically relevant (Gong et al., 2003; Lesné et al., 2006).

Collectively, the data show that ACU193 binds AβOs with 650-fold selectivity versus Aβ monomers (perhaps > 10,000-fold on molar basis), exhibits limited to no plaque binding, and binds to a broad range of synthetic and endogenous low, mid, and higher molecular weight AβOs. Based on these and other data, ACU193 is expected to target therapeutically relevant AβOs effectively within the brain in early AD patients.

## ACU193 Protects Neurons From Amyloid β Oligomer-Induced Synaptic Toxicity

An essential capability of ACU193 is preventing AβOs from attacking synaptic receptors. A series of *ex vivo* studies was carried out to quantify the ability of ACU193 to block or minimize AβO-induced neuronal toxicities.

### ACU193 Prevents Amyloid β Oligomer-Induced Compromise of Synaptic Plasticity

**FIGURE 7:**
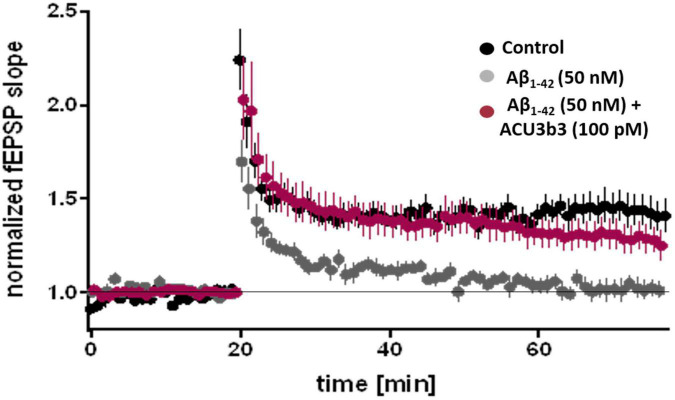
Effects of ACU3B3 and ACU193 on AβO-induced change in LTP (Cline et al., 2019). Addition of 50 nM Aβ42 to brain slice media forms AβOs that block normal LTP, however, pre-incubation with 100 pM ACU3B3 prevents LTP disruption. (see Rammes et al., 2011 for methods).

In *ex vivo* studies using the murine hippocampal slice LTP model (Rammes et al., 2011), pre-incubation with 100 pM ACU193 or ACU3B3 (murine precursor of ACU193) can prevent AβO-induced LTP blockage, as illustrated in Figure 7 (Cline et al., 2019). As discussed earlier in section “Two Decades of Profiling Bolsters the Amyloid β Oligomer Hypothesis,” AβO disruption of LTP has been widely documented. LTP blockage prevents synaptic strengthening that is essential for learning and memory, and the ability of ACU193 to prevent AβO-LTP disruption may be a key property for preventing further memory deterioration in early AD patients.

### Prevention of Amyloid β Oligomer-Induced Disruption of Calcium Homeostasis

**FIGURE 8:**
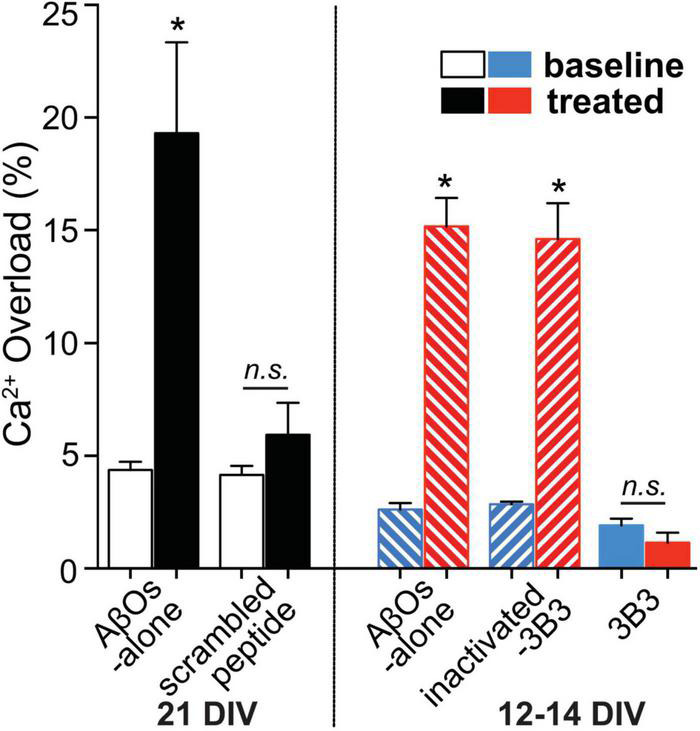
The relationship of AβOs and neuronal calcium homeostasis in the presence and absence of ACU3B3 was studied in primary cultures of transgenic APP-PS1 mouse cortical neurons (Wang et al., 2018). Multiphoton microscopy was used to obtain images of neuronal cultures at 12–14 days *in vitro* (DIV), or 21 DIV. Cortical regions were identified and reimaged before and after topical applications of AβOs to allow comparison of resting calcium within the same neuronal compartments. After baseline calcium measurement, the cultures were treated with antibody-immunodepleted AβOs (1 mL of 3 nM AβOs with 9 μg of antibody) or AβO alone (150–180 pM) for 45 min. The cultures were then re-imaged in the same areas in the dish. AβO assembly of 1–3 nM Aβ42 monomer in cell culture media was carried out at 37°C (Rammes et al., 2011). n.s., non significant; *Signifies a *P*-values of < 0.05.

Exposure to ACU3B3 prevents AβO-induced calcium overload in cortical neuronal cultures (Figure 8; Wang et al., 2018). Disruptions in calcium homeostasis that cause cellular dysfunction have been implicated in a number of disease states, including myocardial infarction and stroke. Further, AβOs have been shown to cause disruption of calcium homeostasis, and thus, restoration of intracellular calcium to normal levels could serve as a functional indicator of treatment effect in AD. Multiphoton microscopy was used to examine the relationship of AβOs and neuronal calcium homeostasis *in vitro* (Figure 8). Direct application of AβOs elicited calcium elevations in cortical neuronal cultures. Prior exposure to ACU3B3 prevented this calcium elevation (Figure 8). These results demonstrate that AβOs induce elevated concentrations of intracellular neuronal calcium and that ACU3B3 prevented the AβO-induced calcium overload.

## *In vivo* Pharmacology

Independent behavioral studies were performed to characterize the *in vivo* central pharmacologic activity of peripherally administered ACU3B3. The behavioral effects observed in these studies indicate that ACU3B3 crosses the blood–brain barrier to engage the target, resulting in behavioral improvements in the transgenic AD model mice.

### Behavioral Studies of Alzheimer’s Disease Mouse Models After Sub-Chronic ACU3B3 Treatment Administered Pre- and Post-Plaque Accumulation

**FIGURE 9:**
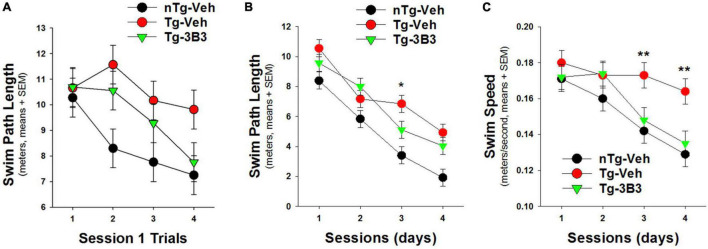
ACU3B3 treatment in 9- to 10-month old APPSL mice (*n* = 10/group) (Faizi et al., 2012) improves performance on the first day of water maze training (**A**; *p* = 0.057), decreases swim path length (**B**; *p* = 0.034), and reverses a swim speed abnormality (**C**; *p* < 0.02). **Signifies a *P*-values of < 0.01.

In a blinded study (Figure 9) conducted by QPS Austria GmbH, plaque bearing 9- to 10-month-old, female, APP/SL transgenic (Havas et al., 2011; Faizi et al., 2012) and non-transgenic mice were administered ACU3B3 (20 mg/kg) or vehicle (PBS) by intraperitoneal (IP) injection once a week for 4 weeks. The last injection was given 24 h before the first water maze training trial. Prior to treatment, transgenic mice demonstrated behavioral deficits compared to non-transgenic mice, but as shown in Figure 9B, significantly improved their performance across testing sessions. On the first training day, ACU3B3 treatment improved swim path length in transgenic mice (Figure 9A), and this effect was significant (*p* < 0.05) after exclusion of outliers. ACU3B3 also improved swim path length performance in session 3 trials (Figure 9B), and restored swim speed in transgenic mice (*p* < 0.02) to the same level as in wild-type mice (Figure 9C).

**FIGURE 10:**
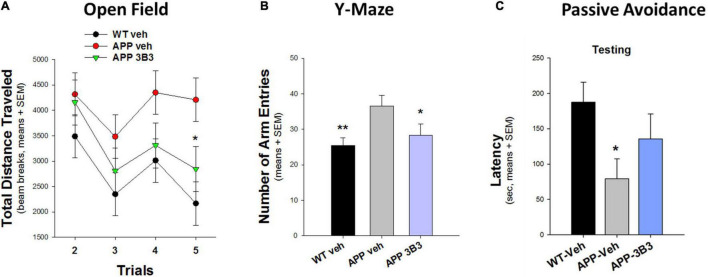
ACU3B3 treatment at 20 mg/kg in 5- to 7-month-old Thy1-hAPP/SL mice (*n* = 13–14/group, means + SEM). **(A)** Open field total distance measurement, APP-Veh vs. APP-3B3, *p = 0.029. **(B)** Y-maze arm entries, APP-Veh vs. APP-3B3, *p = 0.045; APP-Veh vs. WT-Veh, **p = 0.007. **(C)** Passive avoidance latency, APPSL-APP3B3 vs. APPSL-Veh trended for drug effect, but was not statistically significant (Dodart et al., 2014).

In another blinded study (Figure 10) conducted at Stanford University (Dodart et al., 2014), the hyperactivity phenotype of 5- to 7-month-old Thy1-hAPP/SL transgenic mice in the Open Field and Y-Maze tests was also significantly reduced after 4–5 weeks of ACU3B3 treatment (20 and 30 mg/kg, weekly). Prior to dosing, Thy1-hAPP/SL mice showed increased activity in the activity chamber compared to wild-type mice. After ACU3B3 treatment, Thy1-hAPP/SL mice activity fell to a level comparable to wild-type mice, particularly activity in the center of the test arena (Figure 10A). Similar effects of ACU3B3 were found with changes in Y-maze behavior (Figure 10B) and passive avoidance (Figure 10C).

Additional studies were conducted at the Gladstone Institute of Neurological Disease in young hAPP/J20 mice, which express APP carrying the Swedish and Indiana mutations (Mucke et al., 2000). Animals, ages 2–5 months were dosed with ACU3B3 to evaluate its efficacy prior to detectable amyloid deposition. Treatment intervention preceded the appearance of measurable levels of amyloid plaques as well as the progressive decrease in synaptic markers, microglia activation and tau hyperphosphorylation (Shankar and Walsh, 2009) but coincided with the appearance of measurable levels of AβOs (Johnson et al., 2020). ACU3B3 significantly improved behavioral deficits measured in Open Field, Y-Maze, Plus Maze, and Morris Water Maze tests (Cline et al., 2019; Ma et al., 2019), supporting the AβO selectivity of ACU3B3 and its potential for intervening prior to a stage where synaptic loss and the underlying behavioral impairment and memory loss becomes irreversible.

Taken together, the extent of behavioral studies conducted in two different APP transgenic lines with different AD phenotypes and at different stages of disease progression (notably pre- and post-plaque accumulation) provides a comprehensive understanding of the pharmacological activity across age groups. The beneficial behavioral effects observed in animals treated with ACU3B3 indicate that the antibody effectively crosses the blood–brain barrier to engage target AβOs. The dose range selected in the behavioral studies is also representative of the dose range evaluated in the ACU193 phase 1 clinical trial.

## Discussion: The Right Therapeutic to Intercept Amyloid β Oligomers, the Relevant Alzheimer’s Disease Neurotoxins

This focused account highlights the target and the essential properties of ACU193, the first AβO-selective immunotherapeutic clinical candidate for AD. This account also provides a detailed profile of AβOs as the instigating AD neurotoxins. Because AβOs exert their memory-compromising effects at such low concentrations, amidst much higher concentrations of Aβ monomer and extensive amyloid plaques, the attributes required of an effective therapeutic candidate are formidable. While inhibition of secretases was certainly a rational approach to treatment of AD, this approach was unfortunately not successful. The monoclonal antibodies targeting amyloid plaque and perhaps showing some clinical efficacy are all burdened with ARIA and the need for additional MRI monitoring. ACU193 has many essential properties required of a successful therapeutic, and its unique property is its selective binding to AβOs. The investigation of ACU193 in clinical trials in patients with early AD will provide an important test of the amyloid oligomer hypothesis.

## Author Contributions

GK organized and wrote the first draft of the manuscript. ES, EC, and JJ contributed to one or more sections of the manuscript. All authors contributed to manuscript revision, read, and approved the submitted version.

## Conflict of Interest

GK is a co-founder, shareholder and compensated scientific advisor of Acumen Pharmaceuticals. EC is an employee of Acumen Pharmaceuticals, Inc. JJ is an employee, shareholder, and option holder of Acumen Pharmaceuticals, Inc. ES is an employee (Chief Medical Officer) and option holder of Acumen Pharmaceuticals, Inc. Since 2019, ES has consulted or is consulting for Acelot Inc., Aquestive Therapeutics Inc., Athira Pharma, Inc., Biogen Inc., Cogstate Ltd., Cortexyme Inc., Gates Ventures LLC, Hoffman La-Roche Ltd., Indiana University, LuMind Research Down Syndrome Foundation, Partner Therapeutics Inc., Pinteon Therapeutics Inc., Prothena Inc., Vaccinex, Inc., Washington University (St. Louis), Alzheimer’s Association, Bright Focus Foundation, Huntington Study Group, and Michael J. Fox Foundation. ES is a shareholder of Eli Lilly and Company.

## Publisher’s Note

All claims expressed in this article are solely those of the authors and do not necessarily represent those of their affiliated organizations, or those of the publisher, the editors and the reviewers. Any product that may be evaluated in this article, or claim that may be made by its manufacturer, is not guaranteed or endorsed by the publisher.
